# A Rare Case of High-Grade Undifferentiated Pleomorphic Sarcoma of the Terminal Ileum in a 40-Year-Old Man: Successful Outcome and Prolonged Survival After Surgical Resection and Adjuvant Chemotherapy

**DOI:** 10.7759/cureus.94041

**Published:** 2025-10-07

**Authors:** Anusha Manje Gowda, Navkirat Kahlon

**Affiliations:** 1 Hematology/Oncology, Mary Washington Hospital, Fredericksburg, USA; 2 Hematology and Medical Oncology, Mass General Cancer Center, Dover, USA

**Keywords:** adjuvant chemotherapy, chemotherapy (adriamycin), ifosfamide, small bowel tumors, undifferentiated pleomorphic sarcoma (ups)

## Abstract

Undifferentiated pleomorphic sarcoma (UPS) of the small intestine is an exceedingly rare malignancy associated with a poor prognosis, characterized by a high risk of recurrence and metastasis. The typical age of onset is between 61 and 70 years. Only a few cases of UPS in the terminal ileum have been successfully treated with complete surgical resection, followed by adjuvant chemotherapy, leading to favorable outcomes and prolonged disease-free survival. Currently, there are no standardized treatment guidelines for UPS due to the tumor’s rarity, and the role of adjuvant chemotherapy in the management of UPS in the small intestine remains inadequately explored in the literature. We present the case of a 40-year-old man diagnosed with high-grade UPS of the terminal ileum. The patient underwent successful surgical resection with negative margins, followed by adjuvant chemotherapy with the Adriamycin, ifosfamide, and mesna (AIM) regimen. At his 30-month follow-up, the patient remains disease-free. In conclusion, the complete surgical resection of UPS of the small intestine, combined with adjuvant chemotherapy, can lead to favorable outcomes and long-term disease-free survival.

## Introduction

Undifferentiated pleomorphic sarcoma (UPS), formerly known as malignant fibrous histiocytoma (MFH), accounts for less than 1% of all adult tumors and approximately 28% of all soft tissue sarcomas, typically occurring in the extremities and retroperitoneum [1]. UPS of the small intestine is exceptionally rare, often linked to a poor prognosis because of its high tendency to recur and metastasize [2]. In 2013, the World Health Organization classification of soft tissue tumors was revised, and the term “malignant fibrous histiocytoma (MFH)” was replaced by the term “undifferentiated pleomorphic sarcoma” [3]. The peak age of incidence is around 61-70 years of age; it commonly presents in the extremities, with a lower incidence of occurrence in the abdominal cavity and retroperitoneum [4,5]. The rate of the local recurrence of the tumor is 44%, and the incidence of metastasis is 42%; peritoneal metastasis is the most common site of spread, followed by lymph node metastasis, intraperitoneal solid organ metastasis, and lung metastasis [4,5]. The prognosis of intra-abdominal UPS is poorer than that in the extremities due to a delay in diagnosis and a lack of effective treatment options [6]. Tumor cells have marked cytological and nuclear pleomorphism; the presence of bizarre giant cells is common, often admixed with spindle cells and rounded histiocyte-like cells in varying proportions [7]. UPS cells are frequently positive for vimentin, actin, cluster of differentiation 68 (CD68), α1-antitrypsin, and α1-antichymotrypsin [8].

Most of the existing knowledge comes from isolated case reports and small case series. There are very few successfully treated cases of UPS reported, involving the terminal ileum, with prolonged disease-free survival. There is limited literature available on the role of adjuvant chemotherapy in UPS of the small intestine. We present a unique case of the rare occurrence of high-grade UPS in the terminal ileum of a 40-year-old man, which is significantly younger than the typical onset age, and the successful outcome following surgical resection and adjuvant chemotherapy with the Adriamycin, ifosfamide, and mesna (AIM) regimen. The patient remains disease-free at his 30-month follow-up.

## Case presentation

A 40-year-old man with no significant medical history was hospitalized after he presented with a three-month history of progressively worsening generalized abdominal pain, melanotic stools, and alternating episodes of diarrhea and constipation. He also reported exertional dyspnea and persistent fatigue over the same period. The patient’s family history included colon cancer in two maternal second cousins and a maternal great-uncle, as well as skin cancer in his maternal grandmother. His personal history included cigarette smoking (half a pack per day for 10 years, quitting 10 years before presentation) and daily alcohol consumption, which he had stopped two months before presenting.

Laboratory evaluation at presentation (Table 1) was notable for leukocytosis with a white blood cell count of 17.8 K, anemia with hemoglobin of 7.6 g/dL, thrombocytosis with platelets of 761 K, and iron studies revealing iron saturation of 5% and ferritin of 148 ng/mL.

**Table 1:** Laboratory findings at presentation WBC, white blood cell; MCV, mean corpuscular volume; RDW, red cell distribution width; CEA, carcinoembryonic antigen

Laboratory test	Result	Units	Reference range
WBC	17.8	K/μL	4-11.0 K/μL
Hemoglobin	7.6	g/dL	13.0-17.0 g/dL
Hematocrit	25	%	40%-50%
MCV	78	fL	80-94 fL
RDW	17	%	11%-15%
Platelet count	761	K/μL	130-400 K/μL
Neutrophil count	13.89	K/μL	1.6-7.5 K/μL
Lymphocyte count	2.04	K/μL	1.4-7 K/μL
Monocyte count	1.45	K/μL	0.6-4.5 K/μL
Eosinophil count	0.28	K/μL	0-0.6 K/μL
Albumin	3.4	g/dL	4.1-5.1 g/dL
Iron saturation	5%	%	20%-50%
Ferritin	148	ng/mL	27-300 ng/mL
Vitamin B12	499	pg/mL	239-931 pg/mL
Folate	9.81	ng/mL	>2.75 ng/mL
Creatinine	0.6	mg/dL	0.7-1.2 mg/dL
CEA	<0.31	ng/mL	<3.0 ng/mL

**Figure 1:**
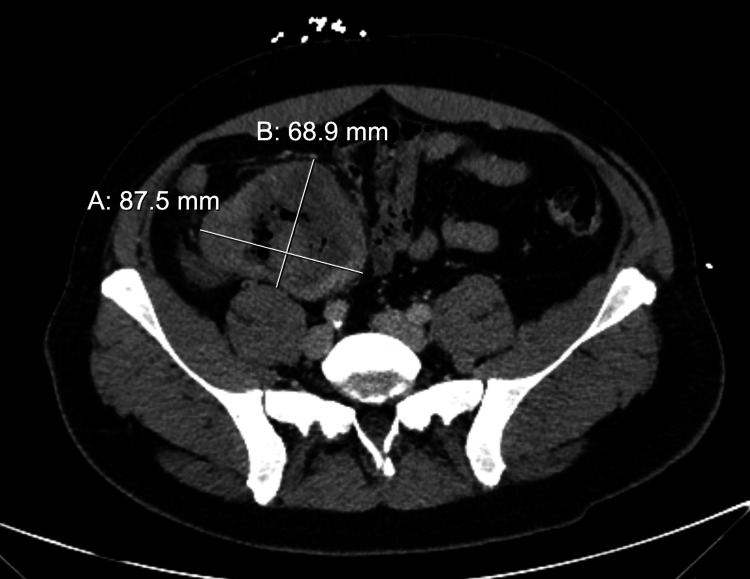
Computed tomography of the abdomen and pelvis with intravenous contrast, with coronal view showing a large 12.1 × 6.9 × 8.8 cm mass arising from the terminal ileum with multiple enlarged regional lymph nodes

A contrast-enhanced computed tomography (CT) pulmonary angiogram was performed to evaluate his exertional dyspnea. The study showed no evidence of pulmonary embolism or metastatic disease and was otherwise unremarkable. A CT of the abdomen and pelvis with intravenous contrast (Figure 1) revealed a large mass arising from the terminal ileum with multiple enlarged regional lymph nodes.

Colonoscopy obtained during hospitalization revealed a necrotic-appearing partially obstructive mass in the terminal ileum. Colonoscopy-guided biopsy of the mass showed necrotic material, along with lymphoid hyperplasia. There was insufficient viable cellular material for further workup. At that time, the differential diagnosis included a solid tumor or lymphoma originating from the terminal ileum, as well as a complex abscess involving the same region. His symptomatic anemia, manifested by progressively worsening exertional dyspnea, was attributed to the underlying disease with a likely contribution from iron deficiency. He received intravenous iron infusions for his iron deficiency anemia.

A week later, he underwent a right-sided hemicolectomy with the resection of the mass, along with lymph node dissection. There was noted to be a large, approximately 15-20 cm right lower quadrant abdominal mass that was partially adherent to the peritoneum of the right lower quadrant. The mass seemed to originate in the terminal ileum and involve the cecum. There were palpable, suspicious lymph nodes within the mesentery of the terminal ileum; peritoneal surfaces were normal. Approximately 18-20 inches of small intestine were resected to achieve a complete resection of the tumor. A portion of the small bowel revealed a large mass measuring 8 cm longitudinally and 12 cm circumferentially. The mass was nearly completely circumferential and was located within 2 cm of the ileocecal valve. A large perforation was present, measuring 4 cm in diameter. Adjacent to the perforation, the mass invaded an adherent loop of bowel. The area of invasion within the adherent loop measured 0.8 cm in diameter. Sectioning the mass revealed invasion through the wall and extends approximately 4 cm into the surrounding fat. The remainder of the bowel showed no additional abnormalities.

**Figure 2:**
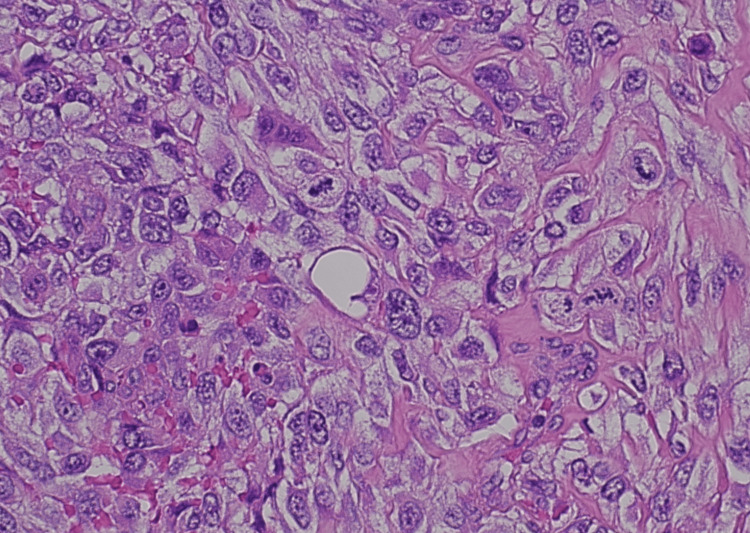
Histopathology slide stained with hematoxylin and eosin (H&E) under high power (400× total magnification) showing high-grade malignant cells with associated multinucleated giant cells and prominent areas of necrosis with spindle cell appearance

Pathology revealed high-grade undifferentiated pleomorphic sarcoma (grade IV). Hematoxylin and eosin staining under high power showed high-grade malignant cells with associated multinucleated giant cells and prominent areas of necrosis with spindle cell appearance (Figure 2). The tumor involved the mucosa, submucosa, muscularis propria, and surrounding fat; there was an invasion of the adjacent right peritoneum. Margins were negative. Twenty-seven mesenteric lymph nodes were removed and were negative for metastatic disease. There was no evidence of macroscopic tumor perforation and lymphovascular invasion. Overall, the features were consistent with high-grade undifferentiated pleomorphic sarcoma (UPS) with a pathological stage of T4N0M0. Next-generation sequencing results showed PDGFRB D850V, tumor mutation burden (TMB) was not high, microsatellite was stable, and tumor proportion score (TPS) was 25%.

About five weeks later, after achieving successful recovery from the surgery, he was initiated on adjuvant systemic therapy with six cycles of Adriamycin and ifosfamide, along with mesna (AIM regimen). He successfully completed six cycles of chemotherapy and recovered well from this. He developed chronic diarrhea following surgical resection, which has been controlled with anti-diarrheal medications. After the completion of adjuvant systemic therapy, he underwent serial surveillance imaging every four months. He is doing well without any recurrence at 30 months since diagnosis. Leukocytosis, anemia, and thrombocytosis have resolved, and all his laboratory parameters are within normal limits at his 30-month follow-up.

## Discussion

The mainstay of treatment of UPS involves the complete resection of the tumor, with a wide resection of margins and en bloc regional lymph node dissection [9]. R0 resection (complete resection) with clear surgical margins is a favorable prognostic factor for overall survival and local recurrence-free survival [10]. There is no consensus on the role of adjuvant chemotherapy and/or radiation therapy. In the phase III trial the European Organisation for Research and Treatment of Cancer (EORTC) 62012 study, patients with UPS had improved tumor response and overall survival with doxorubicin-ifosfamide compared to single-agent doxorubicin (odds ratio {OR} of 9.90 and 95% CI of 1.93-50.7 and hazard ratio {HR} of 0.44 and 95% CI of 0.26-0.79, respectively) [11]. Young, fit patients with poorly differentiated grade III tumors benefited the most from doxorubicin-ifosfamide [11]. In the neoadjuvant setting, Adriamycin + ifosfamide is the regimen of choice in treating UPS, as gemcitabine + docetaxel was not associated with better disease-free survival or overall survival when used in the neoadjuvant setting [12]. Chemotherapy improves outcomes and overall survival in patients with vascular or lymphatic infiltration or nodal involvement [13,14]. On the contrary, few reported cases of primary UPS of the ileum that have been treated with surgical resection alone, without adjuvant chemotherapy, have shown favorable outcomes [15]. As our patient had a resectable tumor, the multidisciplinary consensus was to proceed with surgical resection, followed by adjuvant Adriamycin and ifosfamide to reduce the risk of recurrence and metastasis, given the patient’s young age, good performance status, and diagnosis of grade IV, poorly differentiated high-grade UPS. Mesna was added to chemotherapy as per the protocol to reduce the risk of ifosfamide-induced hemorrhagic cystitis.

Other cases of UPS of the small bowel, with the use of adjuvant chemotherapy, resulting in longer disease-free survival, have been reported [13,16-18]. Zhou et al. reported a 69-year-old patient with UPS of the jejunum presenting with intestinal obstruction. The patient underwent laparoscopic partial small intestinal resection, with no signs of intra-abdominal metastasis. Postoperative chemotherapy and radiotherapy were administered, and the patient remained alive without recurrence or metastasis at four months of follow-up [2].

Kawashima et al. detailed a case involving primary UPS of the descending colon. The patient underwent surgical resection and adjuvant chemotherapy and achieved long-term disease-free survival at a seven-year follow-up [16]. Katsourakis et al. reported a case of UPS arising in the jejunum, treated with complete surgical resection, followed by adjuvant chemotherapy with gemcitabine. The patient experienced a recurrence-free interval of two years but eventually died due to disease recurrence [13].

Recurrence with gastrointestinal UPS can occur years after initial diagnosis and treatment. Froehner et al. describe a case of primary UPS in a postoperatively adherent intestinal loop. The patient recovered well after surgical resection; however, they developed a recurrence at nine and a half years after surgery with a solitary recurrent tumor that appeared in the urinary bladder, which progressed rapidly [19]. There is a need for the long-term surveillance of patients with this diagnosis.

Based on the review of cases reported thus far, patients diagnosed at an earlier stage, without lymph node or metastases, who underwent surgical resection with negative margins appeared to have better outcomes. The majority of the patients who received adjuvant therapy with chemotherapy or radiation therapy also appeared to have a longer survival.

## Conclusions

Undifferentiated pleomorphic sarcoma (UPS) of the ileum is extremely rare and typically associated with a poor prognosis. Our patient underwent successful surgical resection with negative margins, followed by adjuvant chemotherapy with Adriamycin and ifosfamide, and remains disease-free at a 30-month follow-up. Prolonged disease-free survival in cases of small intestinal UPS is seldom reported. This case is among the few demonstrating extended survival following adjuvant chemotherapy, an area where clinical data remain limited. As such, adjuvant chemotherapy should be considered in younger, fit patients following complete surgical resection, as it may reduce the risk of recurrence. Additionally, this case underscores the importance of including UPS in the differential diagnosis of small bowel tumors. While the outcome in this case is encouraging, we acknowledge the limitations of drawing conclusions from a single case with a relatively short follow-up period and recognize that late recurrence remains a possibility. Long-term surveillance is therefore essential.
